# Biophysics of Cell-Substrate Interactions Under Shear

**DOI:** 10.3389/fcell.2019.00251

**Published:** 2019-11-08

**Authors:** Neha Paddillaya, Ashish Mishra, Paturu Kondaiah, Pramod Pullarkat, Gautam I. Menon, Namrata Gundiah

**Affiliations:** ^1^Centre for Biosystems Science and Engineering, Indian Institute of Science, Bangalore, India; ^2^Soft Condensed Matter Group, Raman Research Institute, Bangalore, India; ^3^Department of Molecular Reproduction, Development and Genetics, Indian Institute of Science, Bangalore, India; ^4^The Institute of Mathematical Sciences, Chennai, India; ^5^Homi Bhabha National Institute, Mumbai, India; ^6^Department of Physics, Ashoka University, Sonepat, India; ^7^Department of Mechanical Engineering, Indian Institute of Science, Bangalore, India

**Keywords:** focal adhesions, stress fibers, mechanotransduction, shear stress and devices, biophysical models, adhesion strength

## Abstract

Cells adhere to substrates through mechanosensitive focal adhesion complexes. Measurements that probe how cells detach from substrates when they experience an applied force connect molecular-scale aspects of cell adhesion with the biophysical properties of adherent cells. Such forces can be applied through shear devices that flow fluid in a controlled manner across cells. The signaling pathways associated with focal adhesions, in particular those that involve integrins and receptor tyrosine kinases, are complex, receiving mechano-chemical feedback from the sensing of substrate stiffness as well as of external forces. This article reviews the signaling processes involved in mechanosensing and mechanotransduction during cell-substrate interactions, describing the role such signaling plays in cancer metastasis. We examine some recent progress in quantifying the strength of these interactions, describing a novel fluid shear device that allows for the visualization of the cell and its sub-cellular structures under a shear flow. We also summarize related results from a biophysical model for cellular de-adhesion induced by applied forces. Quantifying cell-substrate adhesions under shear should aid in the development of mechano-diagnostic techniques for diseases in which cell-adhesion is mis-regulated, such as cancers.

## Introduction

Cells encounter mechanical forces through their contacts with other cells in tissue as well as from flows in the vasculature. They respond to these forces through multiple levels of feedback, often altering their shape and orientation in response (Schwarz and Gardel, 2012). For example, adherent endothelial cells elongate in the flow direction when exposed to flows that exert forces on them (Ohashi and Sato, 2005). This change of shape is accompanied by a similar alignment in the underlying actin cytoskeleton (Galbraith et al., 1998). Cell adhesion sites reorganize, and the cell cytoskeleton restructures when cells change shape, largely by cross-linking actin in a space-dependent way that alters the local fluidity of the cytoplasm (Levesque and Nerem, 1985). The extravasation of leukocytes from the circulatory system toward a site of inflammation provides another example of the importance of cell adhesion processes and their interaction with flows (Resnick et al., 2003). During cancer metastasis, cancerous cells encounter forces from fluid flow in the interstices between cells in the tumor tissue as well as from flows in the blood (Wirtz et al., 2011).

Focal adhesions (FA), key sites of transmembrane integrin clustering, mediate intracellular force transmission through dynamic mechano-sensitive complexes (Hynes, 2002). These complexes are connected, both mechanically and through biochemical signaling pathways, to the cytoskeleton (Baratchi et al., 2017). The generation of internal cytoskeletal tension, and the signaling cascades that result, underlie cell-substrate interactions (Sawada et al., 2000). Nascent adhesions formed by cells on substrates generally undergo maturation or turnover associated with the recruitment and assembly of actin (Oakes et al., 2014). This maturation requires tensional force, mediated through inactivation of focal adhesion kinase (FAK), the phosphorylation of Src and p190RhoGAP to decrease the activity of Rho and Rho kinase (ROCK) in cells, the recruitment of Rac and other protein complexes to the adhesion sites and a decrease in the local myosin contractility (von Wichert et al., 2003; Broussard et al., 2008). Mature adhesions are transformed into smaller regions through paxillin de-phosphorylation and changes in integrin density (Zamir et al., 1999). FA turnover thus depends on the rates of association and dissociation of molecules at adhesion sites. These sites therefore not only serve as combinatorial sites for differential signaling, but also regulate cellular behaviors (Oakes et al., 2014).

Signaling pathways related to integrins and receptor tyrosine kinases in the FA complex have a network of complex connections that regulate cell anchorage and proliferation. Integrins are co-opted in the cancer cell niche, where they significantly dysregulate adhesion, leading to cell colony expansion (Plantefaber and Hynes, 1989). Transformed cells exhibit poor adhesion to fibronectin rich substrates, have altered morphologies, and show relatively disorganized cytoskeletal constituents (Winograd-Katz et al., 2014). Integrin signaling is essential in pro-survival cell response to chemotherapy, radiotherapy, and resistance to targeted therapeutic agents (Cooper and Giancotti, 2019).

A better understanding of cell adhesion under mechanical cues may help identify highly metastatic cells within a tumor cell population. Being able to quantitatively describe adhesion processes could lead to a biophysical, as opposed to biochemical, marker for cancer cell metastasis (Fuhrmann et al., 2017). However, quantifying cell-substrate adhesion strength is challenging for several reasons. First, there are no commercial instruments to perform such experiments. Second, deadhesion data are not easily converted to absolute adhesion parameters because cells assume complex shapes. They also have varying spread area and their FAs distributions may be further altered under applied stresses. Fluid shear-based deadhesion experiments to quantify the critical shear stress for cell detachment offer a distinct advantage in measuring the attachment strength of cells to substrates (Holle and Engler, 2011; Maan et al., 2018).

In this article, we discuss key signaling molecules involved at the integrin and cytoskeletal levels, specifically in the context of FA in cancer metastasis. We provide a comparison of various methods to quantify cell-substrate adhesions. Finally, we discuss biophysical models that quantify the strength of interactions between the cell and substrate. Such studies on the dynamics of cells under shear should aid in mechano-diagnostic approaches to assess integrin-substrate interactions. They may also help in the development of therapeutic agents that target the role of integrins in cancer metastasis.

## A Survey of Cell Adhesion

**Figure 1:**
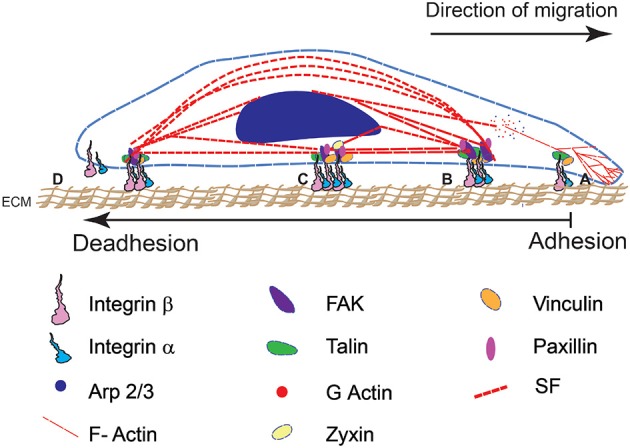
A schematic representation of cell adhesion and deadhesion events during migration and remodeling shows four major steps (from right to left). The direction of migration and sequence of adhesion-deadhesion steps are indicated. **(A)** FA assembly on the leading end of the cell begins with αβ integrin subunits binding to RGD domains in the ECM substrate. **(B)** This leads to integrin clustering and the formation of a mature FA complex. **(C)** Cytoskeletal interactions with the FA result in the development of contractile forces. **(D)** Mature FA at the rear end disintegrate that results in cell retraction.

The transduction of bidirectional mechanical signals from the extracellular matrix (ECM) to the cell cytoskeleton is primarily mediated via focal adhesions (Figure 1). Integrins, comprising the FA complexes, are heterodimeric, consisting of α and β subunits. They form a large family of 24 transmembrane cell surface receptors that connect the ECM to intracellular constituents, provide polarity, and help generate the required tractions for cellular adhesion, motility and invasion during metastasis (Hynes, 2002; Sun et al., 2016). Differences in integrin attachments lead to alterations in adhesion, cytoskeletal organization, and cellular morphology in transformed cells.

A balance in the expressions of β1 and β3 integrins determines tensional homeostasis in a cell (Milloud et al., 2017). β1 integrin deletion causes a decrease in contractile forces whereas deletion of β3 integrin induces an increase in the activation of β1 integrin. These lead to modifications in cell shape and a corresponding change in the spatial distributions of cellular tractions (Milloud et al., 2017; Oria et al., 2017). Tension generated by the cytoskeleton is used to sense the mechanical properties of the ECM. In turn this influences cytoskeletal organization and cell behaviors (Discher et al., 2005; Gardel et al., 2010; Kulkarni et al., 2018a).

**Figure 2:**
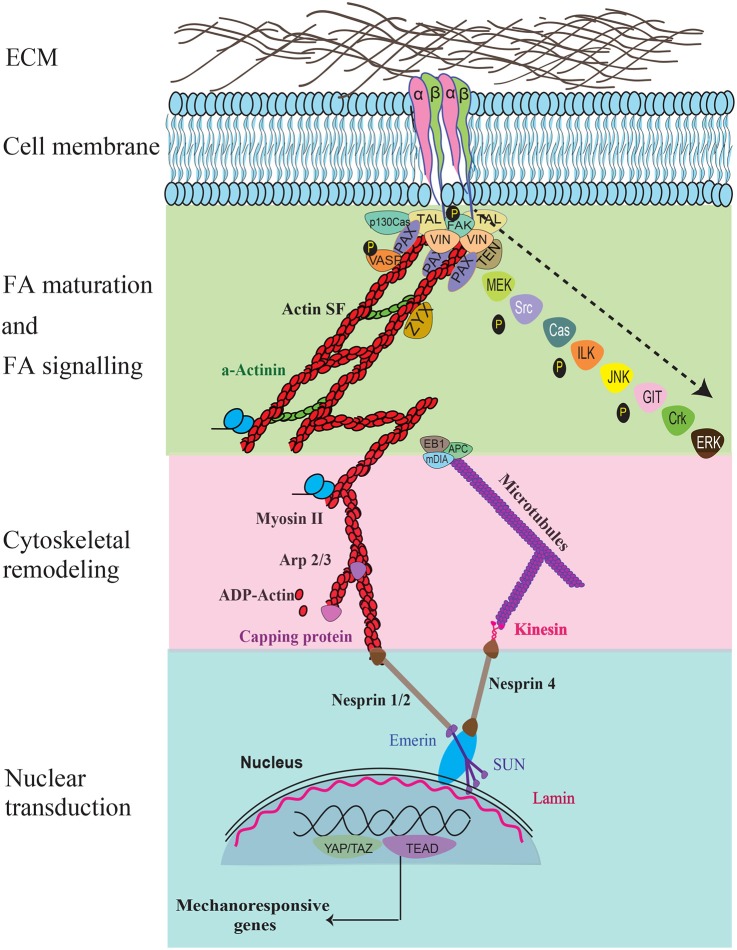
The key force sensing and signaling components during mechanosensing and their links to the nucleus are illustrated. Specific signaling molecules that are temporally recruited during FA maturation, FA signaling, cytoskeletal remodeling, nuclear transduction, and gene response are shown. Recruitment of the various molecules that occur temporally during FA maturation and signaling are indicated using an arrow.

Cellular mechanotransduction in response to external mechanical cues mainly consists of conformational changes in proteins connecting intracellular proteins in the cytoskeleton to the ECM through the FA complex (Figure 2). Tyrosine phosphorylation is a key signaling component which triggers integrin binding to ligands in the external environment and regulates cell adhesion (Kornberg et al., 1991). Integrins alter their conformational state following binding and can shift between low to high affinity states. Binding of α5β1 integrin to fibronectin behaves as a “catch bond” that strengthens in response to applied forces (Kong et al., 2009; Strohmeyer et al., 2017). Substrate stiffness sensing and cell dynamics are dominated by FA complexes in combination with stress fiber contractility.

FA proteins such as talin, vinculin, and p130Cas have hidden binding sites that are exposed during mechanical loading (Grashoff et al., 2010; Kumar et al., 2016; Gauthier and Roca-Cusachs, 2018; LaCroix et al., 2018). Talin plays a key role in cellular adhesion due to its interactions with the cytoplasmic domains in β-integrins and three actin binding sites (Tadokoro et al., 2003; Goult et al., 2018). The binding of talin to integrins results in unfolding of talin and a corresponding strengthening of the adhesion complex. Haining et al. (2016) used single-molecule atomic force microscopy to show that increased force causes greater talin unfolding which exposes cryptic vinculin binding sites and promotes vinculin binding to F-actin (Humphries et al., 2007; Carisey et al., 2013; Yao et al., 2016). Cells under stretch show talin reinforcement and increased acto-myosin contractility leading to FA maturation (Deakin et al., 2012; Thievessen et al., 2013; Elosegui-Artola et al., 2016; Zacharchenko et al., 2016). Together, these increase vinculin recruitment and facilitate the formation of mature and stable FAs. The exact mechanism for talin mechanosensitivity is still under investigation.

Nascent adhesions show increased levels of phosphotyrosine, paxillin, and vinculin accompanied by relatively low levels of tensin in FA regions. Focal adhesion kinase (FAK) recruitment to FA sites results in translocation of Yes-associated protein (YAP) to the nucleus, accompanied by changes in cellular activity (Lachowski et al., 2018). The recruitment of additional proteins on the cytoplasmic side link the integrins to actin through regulation of the Rho/ROCK pathway (Parsons et al., 2010). The binding of actin to myosin II and α-actinin is accompanied by crosslinking and the organization of filamentous actin networks into stress fibers that mediate the cytoskeletal tension in the nucleus and regulate gene/protein responses (Geiger et al., 2001). In contrast, mature FAs contain high levels of tensin and almost no phosphotyrosine (Zamir et al., 1999). Traction studies show that the force applied by a cell is closely linked to the FA assembly at the substrate (Balaban et al., 2001). TFM (Balaban et al., 2001), magnetic tweezer (Walter et al., 2006), and optical tweezer (Schwingel and Bastmeyer, 2013) studies show transformation of transient early adhesions into mature adhesions under mechanical load.

Given the large number of ways in which forces can influence microscopic mechanisms for cell adhesion, biophysical methods to apply forces to cells are critical to our understanding of the mechanobiology of cell-substrate sensing and regulation. The use of FRET sensors to quantify mechanosensitivity of the proteins coupled with measurements of actin flows and traction force microscopy are promising directions to address the roles of specific molecules in substrate sensing, adhesion, and migration (Kumar et al., 2016). Such studies also provide important directions to characterize the links between cell morphology and contractility in ECM remodeling associated with various pathologies like fibrosis and cancer.

## Cell-Substrate Interactions in Carcinoma Cells

The coordination of FA assembly and disassembly is substantially altered in cancer cells (Kai et al., 2016). Several molecules are involved in fibronectin upregulation and proteolysis of the basement membrane during tumor progression (Gopal et al., 2017). The composition and mechanical properties of the ECM produced by tumor cells and the host microenvironment change significantly with respect to normal cells. Mutations leading to mis-regulated ECM remodeling are associated with pathologies such as fibrosis and cancer (Cox and Erler, 2011). Differences in integrin attachments lead to altered adhesion as well as changes in cytoskeletal organization and cellular morphology in transformed cells. Specific integrins, such as αvβ3, α5β1, and αvβ6, are highly expressed in some tumors but show significantly less expression in normal epithelial cells (Kren et al., 2007). Many molecules are involved in fibronectin upregulation and proteolysis of the basement membrane during tumor progression (Gopal et al., 2017). Cellular functions such as attachment-detachment, proliferation, migration and invasion also undergo significant changes (Reymond et al., 2012).

FAK/SFK signaling dominates the integrin-mediated mechanotransduction in cells. Integrins αvβ5, αvβ6, and αvβ8 activate TGF-β signaling in carcinoma cells via forces on the latency associated peptide (LAP) (Bianconi et al., 2016; Khan and Marshall, 2016). Integrins α4β1 and α5β1 bind to fibronectin and increase tumor cell migration, invasion, and metastasis (Brooks et al., 2010). Others such as α6β4 bind to laminin to form a signaling complex with Met, HER2, and EGFR to increase cell invasiveness. Shibue and Weinberg (2009) showed that β1-mediated signaling promotes proliferation of metastatic lung cells. Expression of integrin, αvβ3, in breast cancer cells depends on TGF-β2 that activates a transcription factor, Slug, to induce epithelial–mesenchymal transition (EMT) (Desgrosellier et al., 2014). Integrins αvβ3, β1, and β4 are involved in the adhesion of circulating tumor cells to the endothelial cells (Laferriere et al., 2004; Klemke et al., 2007; Reymond et al., 2012). α5β1-dependent adhesions are more stable due to their higher binding energy but also have a longer bond lifetime than integrin αvβ3 integrin (Roca-Cusachs et al., 2009; Kong et al., 2013; Bharadwaj et al., 2017). YAP/TAZ and TGF-β signaling are both linked to tumor invasion and fibrosis in late-stage cancers (Liu et al., 2017).

Knockdown of FAK, Integrin linked kinase (ILK), talin, and zyxin in breast cancer (MCF7) cells leads to enlarged FA and decreased migration (Fokkelman et al., 2016). Highly invasive breast and oral squamous cancer cells exhibit reduced cell-substrate attachment and higher motility (Richard and Pillai, 2010). Phosphorylation of FAK in cancer cells is associated with integrin adhesion dynamics and deregulation of E-cadherin during SRC associated epithelial to mesenchymal transitions (McLean et al., 2005; Sen and Johnson, 2011). Vinculin knockdown cells show elevated cell migrations due to increased paxillin and FAK phosphorylation accompanied with higher turnover in the FA (Mierke et al., 2010). Activation of vinculin in cancer cells via substrate stiffening through PI3-kinase activation and basal membrane invasion promotes tumor progression (Rubashkin et al., 2014).

In other cases, laminin-binding integrins have been shown to promote and inhibit growth (Ramovs et al., 2017). Intermediate filaments (IF) of tumor epithelial cells have a different composition as compared to normal cells. IFs in tumor cells have higher vimentin in contrast to keratin in normal cells; the presence of vimentin is a marker of EMT in mammary tissues (Kokkinos et al., 2007). More recent studies show that translocation of YAP to the nucleus is related to tumor growth and metastasis (Zanconato et al., 2016).

Invasive cancer cells generate higher tractions that may reflect the metastatic state of cancer cells (Koch et al., 2012). The deformability of breast cancer cells has a context dependent response and is linked to a higher expression and orientation of stress fibers in the cytoskeleton (Kulkarni et al., 2018b). Cancerous endothelial cells on fibronectin gels have greater tractions (~100 nN) than healthy EC (~50 nN) (Ghosh et al., 2008). Higher tractions are attributed to the formation of invadopodia through highly localized actin polymerization under the influence of cofillin, Arp2/3, and N-WASP (Yamaguchi et al., 2005).

Higher cellular contractility through activation of Rho GTPases leads to higher forces on the ECM via integrins that may further act as a driving force in cancer progression (Levental et al., 2009). For example, the increased expression of αvβ3 integrin and SRC activation is seen in cancer stem cell like phenotype, resistance to anoikis and increased metastasis in breast and lung cancers (Desgrosellier et al., 2009). Integrins can act both as tumor promotors and suppressors. Integrin α3β1 causes tumorigenesis (Cagnet et al., 2014) whereas α2β1 integrin is a metastasis suppressor in breast cancer (Ramirez et al., 2011).

The higher stiffness of tumors, associated with increased deposition of collagen I and fibronectin, is an easily detectable mechanical feature through palpation and is generally used in diagnosis (Chaudhuri et al., 2014). Stiffening of the ECM occurs via increased collagen cross-linking, associated with lysyl oxidase, and leads to tumor progression (Kirschmann et al., 2002; Erler et al., 2006). Transglutaminase 2 association and its co-localization with fibronectin influences cell-matrix interactions through integrin binding (Zemskov et al., 2006). The ECM composition and anisotropy are directly correlated with prognosis and patient survival (Conklin et al., 2011). Tumors have thicker ECM fibrils that align perpendicular to the tumor boundary as compared to non-oriented ECM fibrils in normal tissues. The ECM stiffness and anisotropy serve as migration tracks for cancer cells and promote invasiveness through generation of differential tension (Kirschmann et al., 2002; Provenzano et al., 2006).

Tumor cells are subjected to tensile stresses, such as pressure from solid tumor formation, and to interstitial shear stress as they enter the vascular or lymphatic system during metastasis. The evolving tumor microenvironment has increased flow rate and high vascular permeability (Wirtz et al., 2011). These biomechanical forces induce signaling from the extracellular environment, through the membrane, into the cytosol and the nucleus. Breast cancer (Polacheck et al., 2011) and glioma cells (Munson and Shieh, 2014) show increased migration within three dimensional *in vitro* cultures due to continuous interstitial fluid flow. Metastasizing primary tumor cells or circulating tumor cells enter the blood vessel and are the most common cause of cancer recurrences (Rejniak, 2016). A fraction of circulating tumor cells (~0.02%) survive to metastasize; others are killed by anoikis, NK cells or forces due to FSS (Massague and Obenauf, 2016; Rejniak, 2016).

Cell deadhesion strength has been shown to be directly proportional to the number of α5β1 integrin bonds formed with fibronectin (Shi and Boettiger, 2003). A single integrin-ligand bond requires a force of ~50–100 pN force to cause bond rupture (Litvinov et al., 2002; Li et al., 2003; Weisel et al., 2003). Boettiger (2007) used a spinning disc device to quantify the cell adhesion strengths for cells attached to ECM coated surfaces. Fuhrmann et al. (2014) used a spinning-disk device to apply force on cell populations and characterized the differences in the adhesion strengths of metastatic mammary epithelial cells. They showed that the cell adhesion strength is useful to delineate highly metastatic cancer cells within a heterogeneous tumor cell population. Other studies show correlations between changes in cellular adhesion and the development of secondary tumors (Fischer et al., 1999; Palmer et al., 2008; Reticker-Flynn et al., 2012).

Cell deadhesion assays are useful methods to quantify differences in cellular adhesion strengths. Such differences may be linked to differences in the FA composition and density. Identifying the key proteins involved in adhesion signaling and linking them with oncogenic events under mechanical stimuli is essential to the development of therapeutics in cancer treatment.

## Mechanobiology of Cells Under Shear

Several cells in the body experience shear stress at various magnitudes. The fluid shear stress (FSS) is given by the product of fluid viscosity and shear rate and is expressed in units of N/m^2^ or dynes/cm^2^. FSS on the endothelium modulates their structure and function through mechanotransduction of the underlying cells (Cunningham and Gotlieb, 2005). Laminar shear induces endothelial cell elongation, suppression of proliferation, redistribution of FA, and modulation in the cytoskeletal organization (Malek and Izumo, 1996). Cell contraction or spreading may also localize FAK (Michael et al., 2009) resulting in changes to the actin organization under shear (Tzima et al., 2001).

**Figure 3:**
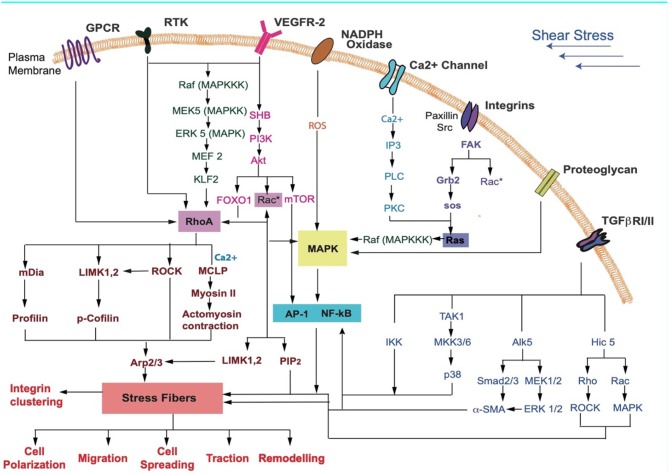
Key receptors in the cell membrane and the various signaling pathways that may be activated during FSS sensing by cells are shown. Receptors activated due to shear stress cause a downstream signaling cascade. These lead to cellular responses such as differentiation, cell cycle arrest, contraction, cytoskeletal alignment, migration, and release of anti-inflammatory markers (Jalali et al., 1998; Gong et al., 2004; Li S. et al., 2005; Zhou et al., 2014; Wilkins et al., 2015; Baratchi et al., 2017; Kunnen et al., 2017; Lee et al., 2017; Chatterjee, 2018).

Perrault et al. (2015) showed that endothelial cells respond to flow with a rapid increase in traction forces and intercellular stresses. Low laminar shear stress, associated with inflammation and atherosclerosis progression, increases cell tractions (Ting et al., 2012). Contractile cytoskeletal forces regulate and facilitate cell elongation in the direction of flow (Lam et al., 2012). Higher tractions are mediated by the Rho-ROCK pathway occur under increased shear (Munevar et al., 2001; Reinhart-King et al., 2003). The endothelium responds with an increase in the cytosolic calcium (Ca^2+^), nitric oxide synthase (eNOS) and nitric oxide production (Fleming and Busse, 2003; Li Y. et al., 2005). High expression of VEGF and VEGFR2 activation are associated with the sensing of fluid shear (dela Paz et al., 2013; Coon et al., 2015). Activation of RTK, Ca^2+^, integrins, GPCRs, and TGF-β, among others, that respond to shear stress result in regulation/activation of downstream effectors such as Rho-Rac (Figure 3). These affect SF contractility and may result in changes to cellular responses such as polarization, migration, cell spreading, traction, and remodeling.

Tumor cells generally experience FSS in the range 0.1–3,000 dyn/cm^2^ (Wirtz et al., 2011). The exposure of cancer cells to FSS activates several signaling pathways that cause remodeling of the actin networks and the FA. The altered adhesion dynamics promotes cell migration through activation of Src (Thamilselvan et al., 2007). Active cytoskeletal remodeling under shear induces specific gene expression that facilitates cell proliferation, differentiation and cancer progression (Olson and Nordheim, 2010). Tumor invasion and metastasis, including cellular adhesion and deadhesion, occurs by FSS that may eliminate circulating tumor cells (CTCs) or promote cell cycle arrest in tumor cells (Fan et al., 2016).

## Biophysical Modeling of Cell Adhesion

### Computing Fluid Shear Stress on Adhered Cells

To quantify cell adhesion in the presence of a flow one must first compute the forces that the flow exerts on the cell. Provided the cell remains adhered, these forces can then be balanced against forces from cell adhesion, thus providing estimates for the strength of adhesion (Katsumi et al., 2005). Fluid flow is described through the Navier-Stokes equation for the local velocity of a so-called Newtonian fluid (Morrison, 2001). This velocity defines a field **u**(**r**, t), since it can be defined at every point in space, at any time. The incompressibility of the flow is imposed by requiring that ∇·**u** = **0**. The Navier-Stokes equation is

(1) $$\begin{array}{r} \rho \left(\frac{\partial }{\partial t}+\text{u}.\nabla \right)\text{u}=-\nabla p+{\eta \nabla }^{2}\text{u} \end{array}$$

where ρ is the density, *p* is the pressure and η the dynamic shear viscosity, a property of the fluid. In addition, boundary conditions must be specified to obtain solutions, since these are differential equations. These require that the normal and tangential components of the velocity vanish at solid surfaces, the no-slip condition. Shear stresses, computable from the velocity field, must obey a stress balance condition across proximate interfaces that are fluid.

The low-Reynolds (*Re*) number limit is appropriate in most cases of cell-scale biological fluid flow. The Reynolds number *Re* = *uL/*η, with *u* = |**u**| a velocity scale, *L* a characteristic length and η the viscosity (Happel and Brenner, 1983). At cellular length-scales, one usually deals with *Re* ≈ 10^–3^ or smaller. In a quasi-steady state, the Navier-Stokes equation in the low *Re* limit reduces to the simpler Stokes equations (Nelson et al., 2004), given by

(2) $$\begin{array}{r} \eta \nabla^{2}u=\nabla \text{p} \end{array}$$

This equation describes the instantaneous force balance. It does not involve time explicitly in this limit, except via boundary conditions that might depend on time. Its solutions are prescribed completely by the imposed boundary conditions.

If the flow is over a flat substrate to which individual cells are attached, the presence of the cell simply enters as a boundary condition on the flow, constraining the velocity field as it approaches the cell surface in specific ways. If the cell presents a solid surface to the flow (if we idealize it as, say, a hemisphere of radius *R* adhered to the surface) the problem is completely specified, since one can assume a no-slip condition for the velocity field at the surface, while also prescribing the flow far away from the adhered cell. All boundary conditions are then available. This formulation ignores several features of the biological system; these are discussed in more detail later.

If we assume that on the scale of the cell, the flow can be approximated by a linear shear flow, this is the problem of creeping flow across a protuberance (Happel and Brenner, 1983). This problem has been studied in several contexts. The central step is the computation of the flow itself, given the Stokes equation and the boundary conditions on the flow. Using this, the stress tensor associated with the flow can be computed. As a next step, the total force and torque exerted on the cell can be extracted by integrating appropriate stress components over the surface. In the case of general shapes, this can be done by integral equation methods. O'Neill (1968) first used an infinite-series solution for the flow over a full sphere in contact with a wall, computing both the force and the torque. Hyman (1972) later considered the corresponding flow over a hemispherical bump but provided only an incomplete solution. Price obtained a solution for the case of hemispherical bumps at which the no-slip condition was satisfied in a calculation that is now used as a benchmark in this limit (Price, 1985). Some years later, Pozrikidis described shear flows over a class of protuberances projecting from a plane surface, determining numerically the forces and torques exerted by a spherical cap or an ellipsoid (Pozrikidis, 1997, 2000). Gaver and Kute studied the effect of flow on a 2D adherent cell in a microchannel (Gaver and Kute, 1998; Hazel and Pedley, 2000). These methods were generalized to the 3D-case. The Gaver and Kute derived formulae were used to benchmark results on flow across adhered cells in microchannels (Couzon et al., 2009). Sugiyama and Sbragaglia later performed a computation similar to that of Gaver and Kute, but for the case in which the cell was modeled as a fluid, recovering earlier results in the limit of infinite viscosity (Sugiyama and Sbragaglia, 2008).

### Model Approaches to Cell Detachment Kinetics

We now discuss models for cell adhesion and describe a recently proposed simple model for how the number of adhered cells vary with the flow (Maan et al., 2018). Such adhesion is primarily mediated by the integrin family of proteins as discussed in earlier sections. The steps for adhesion involve the recruitment of integrins to the cell surface, their activation, and their subsequent coupling to extracellular ligands. At a microscopic level, cell-substrate contacts should include both specific and non-specific interactions, encompassing receptor-ligand interactions, their chemical potentials, their mobility on the membrane, any clustering that arises as a result of signaling, non-specific potentials between cell and substrate, cell deformations and the presence of the glycocalyx. In practice, most if not all of these features are ignored to simplify model building (Sackmann and Smith, 2014; Weikl et al., 2016).

Biophysical models for the effects of force on cell adhesion trace their origins to a pioneering study of antibody-antigen interaction between cell surfaces due to Bell (Bell, 1978; Schwarz and Safran, 2013). Bell described the interactions between antibody and antigen in terms of forward and backward rates for binding and unbinding and their modification by an applied force. The dependence of the barrier height on the applied force was assumed to be exponential. This dependence is consistent with a Kramers-type argument for the rate of barrier crossing between bound and unbound states in the presence of a force. Bell assumed that the load was uniformly shared between attachments. The problem of detachment under force was then studied at a mean-field level.

Consider modeling cell adhesion in terms of a total of *N* potential attachment points that could be bound or unbound with respect to a proximate surface. Each such attachment represents a single focal contact or adhesion. At a given time, a number *N* (*t*) are bound while the remainder *N* – *N* (*t*) are unbound. Each bond can break at rate *k*_*off*_ and the bond can reform at a rate *k*_*on*_. Unbinding in the presence of a force *F* can be assumed to follow *k*_*off*_ = *k*_0_ *exp* (*F*/*F*_0_) where *F*_0_ is a molecule scale force. Note that the off rate is exponentially enhanced by the force. In a mean-field approximation, the mean number of attachments is (Hoffman et al., 2012)

(3) $$\begin{array}{r} \frac{dN\left(t\right)}{dt}=-N\left(t\right){\;k}_{0}\exp\left(Fx_{b}/k_{B}TN\left(t\right)\right)+k_{on}\left(N-N\left(t\right)\right) \end{array}$$

We can simplify this through the introduction of dimensionless times τ = *k*_0_*t*, forces *f* = *F/F*_0_ and rebinding rates γ = *k*_*on*_*/k*_0_. We can further assume that the force is shared equally among all attachments. Then, the solution in steady state can be of several types. Depending on *f*, there can be two solutions (one unstable and one stable, a saddle and a node), a single solution (a saddle node bifurcation point) or no solution at all. The location of the saddle bifurcation is obtained as *f*_*c*_ = *Np ln*(γ/*e*) where the product logarithm is defined as *p* ln(a) from the solution of x exp x = a. Thus, adhesion can only be stable up to a critical force *f*_*c*_. This critical force increases linearly as the rebinding rate γ is increased. If rebinding is forbidden, adhesion is completely unstable.

As is well-known, mean-field descriptions ignore fluctuations (Schwarz and Safran, 2013). These can be incorporated using a one-step master equation for the quantity *p*_*i*_, where *p*_*i*_ is the probability of having *i* adhesion molecules bound at time *t* (van Kampen, 1992; Seifert, 2000; Erdmann and Schwarz, 2004; Liang and Chen, 2011; Hoffman et al., 2012; Schwarz and Safran, 2013). Since binding changes in discrete steps as bonds are broken and reformed, this master equation can simply be written as

(4) $$\begin{array}{r} \frac{dp_{i}}{dt}=r\left(i+1\right)p_{i+1}+g\left(i-1\right)p_{i-1}-\left[r\left(i\right)+g\left(i\right)\right]p_{i} \end{array}$$

The rates that enter this equation can be obtained from the definition of the Bell model. Using the load sharing assumption, $r\left(i\right)\;=\;ie^{\frac{f}{i}}\;$and *g*(*i*) = γ(*N* − *i*). With these rates, the mean first passage time can then be obtained using a result originally due to van Kampen (1992) and Schwarz and Safran (2013). The results from this analysis include a logarithmic dependence of the first passage time on the force for small forces, as well as an exponential dependence at large force. Separating these extremes, a cross-over force value *f*_*c*_ can be defined (Erdmann and Schwarz, 2004). Provided that the number of adhesion molecular is finite, a cell must ultimately deadhere, if one waits long enough, since the first passage time to the deadhered state is finite.

This treatment indicates that two separate regimes might be experimentally relevant. If the mean number of attachment points is instantly equilibrated as the shear stress is ramped in steps, a short-time approximation, is valid (Seifert, 2002; Evans and Calderwood, 2007). In a second limit, one could imagine spending a defined waiting time at each shear stress value. If this waiting time is comparable to the first passage time, the dynamics of detachment cannot be ignored. Further, cooperative effects are important: since the force is shared between bonds (the load sharing assumption), the force on them becomes larger in a non-linear manner as an increasing fraction of bonds are broken.

Modeling the adherent cell as a solid hemisphere provides a way of approximating the force due to a single adhesion complex. For a simple shear flow, with the velocity in the x-direction and the gradient in the z-direction, with shear rate *k* and fluid dynamical viscosity η and thus wall-shear stress σ = η*k*, the total force exerted on the hemisphere is $\vec{F}=13.508\sigma R^{2}\hat{x}$, where *R* the cell radius (Price, 1985). For a numerical estimate we can take *R* ~20 μm (R_HEK_ = 11 μm, R_3T3_ = 24 μm) and values of σ of around 3.5 Pa for HEK and fibroblast NIH 3T3 cell that define the midpoint of the detachment curve for a cell population. This then yields a force-scale of ~19 nN. This estimate is reasonable in comparison to typical experimental values (Dembo and Wang, 1999; Butler et al., 2002; Ambrosi et al., 2009; Couzon et al., 2009; Schwarz and Safran, 2013; Vishavkarma et al., 2014). If we estimate the number of adhesion complexes to be around 40, we can compute that each adhesion complex exerts about 475 pN of force on the substrate that the cell is bound to. This value is reasonable experimentally (Butler et al., 2002).

### An Analytic Description of Cell Detachment Kinetics

A simple set of approximations can be used to relate the detachment curve to the distribution of cell sizes. Gillespie simulations of the master equation indicate that the transition between bound and unbound states is a discontinuous one, although there can be hysteresis in the force value at which it happens. The mean detachment force increases linearly with *N*. As we wait longer at each force value, the threshold shifts to smaller values. However, the step nature of deadhesion continues to be observed, although the average critical force now depends on the waiting-time. The fact that the threshold decreases with increasing waiting time arises because deadhesion involves a barrier crossing process. Given these observations, we can assume that the critical force for the detachment of a cell with *N* attachment points increases linearly with *N, F*_*c*_(*N*) = α*N*.

The applied shear stress σ can be related to the total force *F* experienced by each cell. This will, in general, depend on the shape of the cell and the characteristic length-scales over which the flow is perturbed. The fraction of attached cells observed to remain adhered when the shear stress is increased to σ from zero is defined as Φ(σ), a quantity that should depend on the history of the shear.

We first consider the case where the shear stress is ramped up fast from zero, in the fast-ramping approximation. This ensures that only those cells which are absolutely unstable to detachment are removed when the flow is applied. The derivative $P\left(\sigma \right)=-\frac{d\Phi \left(\sigma \right)}{d\sigma }$ represents the fraction of cells that detach between σ and σ + *dσ*. We wish to calculate *P* (σ) for a set of adhered cells, given in terms of a joint distribution *P* (*N, R*) as

(5) $$\begin{array}{r} P\left(\sigma \right)=\langle \delta \left(\sigma -\sigma_{c}\left(N,R\right)\right)\rangle , \end{array}$$

where the averages, denoted by < >, are over the probability distribution *P* (*N, R*).

The shear stress σ is related to the force exerted on the cell by the flow and the critical value of the shear stress at given *N* and *R* is denoted by σ_*c*_(N, R). Decomposing this joint probability in terms of the conditional probabilities *P* (*N, R*) = *P* (*N*|*R*)*P*(*R*), if we take the conditional probability of having *N* attachment points as slaved to the radius *R*, *P* (*N*|*R*) = δ(*N* − α*R*^*a*^). This means that the distribution of cell sizes determines *P* (σ). The shear force experienced by the cell, as previously derived, is $F_{shear}\;=\;C\overset{∙}{\gamma }\eta R^{2}\;=\;\sigma CR^{2}$. Here *C* is a geometric factor that represents the aspect ratio of the cell, σ the wall stress and *R* is the radius of the circular section of the cell in contact with the substrate. This force is opposed by forces from the FA's: $F_{adhesions\;}=\;Nf\;=\;\alpha R^{a}f\;$. We have assumed that the number *N* of focal adhesions is directly proportional to *R* raised to an appropriate power: If FA's are distributed largely along the perimeter, then *a* = 1. Equating these,

(6) $$\begin{array}{r} C{\overset{∙}{\gamma }}_{c}\eta R^{2}=\sigma_{c}CR^{2}=\alpha R^{a}f \end{array}$$

This provides an estimate for the critical shear stress σ_*c*_ = αR^a−2^*f*/*C*. When *a* = 1, we have $\frac{\alpha f}{CR}=\frac{D}{R}$, where $D=\;\frac{\alpha f\;}{C}$. A convenient analytic form for the distribution of spread cell sizes is the log-normal form (Hammer and Apte, 1992). Finally, Φ(σ), the number of cells remaining as the external stress is ramped up to σ, is obtained as

(7) $$\begin{array}{r} \Phi \left(\sigma \right)=\frac{1}{2}\left[1-erf\left(\frac{ln\left(\frac{\sigma }{\sigma_{0}}\right)}{\sqrt{2s}}\right)\right] \end{array}$$

**Figure 4:**
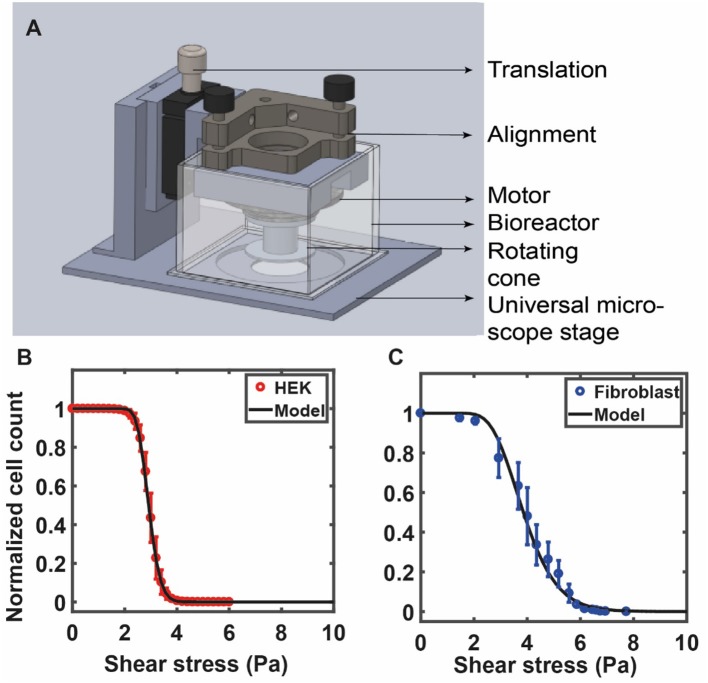
**(A)** The custom fluid shear device used by Maan et al. (2018) is shown in **(A)**. The device is mounted on a microscope for real-time visualization of cells under shear. The device consists of a 1° cone attached to a motor that is used to apply increasing shear stress on cells cultured on a petri dish (see Maan et al., 2018 for details). RPM of the motor is controlled by a computer via a feedback loop. A bioreactor, placed around the device, is used to maintain physiological conditions. The number of adherent cells is counted at each value of shear and is used to quantify the deadhesion strength. **(B)** The curve shows experimental data for HEK cells against the model prediction of Equation (7) with the values of σ_*o*_and *s* obtained from a best-fit analysis: σ_*o*_= 2.91, √2s = 0.16 (HEK). **(C)** Experimental data for 3T3 fibroblast cells against the model prediction of Equation (7) with the values of σ_*o*_ and *s* obtained from a best-fit analysis: σ_*o*_ = 3.85, √2s = 0.35. **(B,C)** Are reprinted with permission from IOP Science, Physical Biology (Maan et al., 2018).

This expression contains fit parameters σ_*o*_ and *s*. The first of these, σ_*o*_, can be directly inferred from the detachment curve. As a test of this formula, we show experimental data for HEK cells in Figure 4B against the model prediction of Equation (7) with the values of σ_*o*_ and *s* obtained from a best-fit analysis. In Figure 4C we show experimental data for 3T3 fibroblast cells against the model prediction of Equation (7) with the values of σ_*o*_ and *s* obtained from a best-fit analysis. As can be seen, the theoretical form provides a good representation of the experimental data (Maan et al., 2018). For the data shown in Figure 4B, these parameters are: σ_*o*_= 2.91, √2s = 0.16 (HEK) and in Figure 4C σ_*o*_ = 3.85, √2s = 0.35 (fibroblasts). The quantity *s* is proportional to the width of the distribution of the number of attachment points across cells, which we assume is equivalent to the distribution of cell radii. Cheung et al. (2009) have used a similar log-normal form to fit their data, but do not correlate this to the distribution of spread areas, treating it as a simple fit form. The derivation provided here is useful because it suggests how such a special form might originate.

More recently, Fuhrmann et al. (2017) have reproduced similar detachment curves in a study of identified Mg^2+^ and Ca^2+^ mediated differences in adhesion strength between metastatic and non-metastatic mammary epithelial cell lines. More refined approaches have to be devised for cases in which the flow is confined in all dimensions. Ambrosi et al. (2009) studied T24 cancer cells adhering to the walls of a microchannel and subjected to an increasing shear flow. To interpret their data, they use an explicit form for forces in the presence of confinement derived by Gaver and Kute (1998), which is

(8) $$\begin{array}{r} \overset{\to }{F}=24\eta \gamma^{2}\frac{Q}{w}\frac{3.19+0.65\gamma +4.34\gamma^{2}}{{\left(1-\gamma^{2}\right)}^{\frac{5}{2}}}\hat{x} \end{array}$$

where η is the fluid viscosity, *R* the cell radius, *Q* the flow rate, and γ = *R/h* is the degree of confinement, with *h* the channel height. The case of the cell-free situation yields the following result for the shear stress at the wall

(9) $$\begin{array}{r} \sigma_{zy}=\frac{6\eta Q}{wh^{2}} \end{array}$$

which can be substituted in the relation above. They find that the cell resists the increasing flow, until a critical stress is reached. This critical stress, if assumed to be a product of the number of adhesion sites with their strength, can be used to calculate a range of values for adhesion parameters for different cell types.

Models for cell behavior in the vascular microenvironment include adhesive dynamics (AD) simulations (Pozrikidis, 2003; Hammer, 2014). The motivation of such simulations is to predict how adhesiveness depends on factors such as shear rate and viscosity. Individual molecular bonds are modeled as compliant springs. The cell is modeled as a rigid spherical particle covered with a random distribution of adhesion molecules. The endothelial cell wall contains counter-receptor molecules. The model assumes that bonds randomly form between adhesion molecules of the cell and wall counter-receptors. Depending on the instantaneous force loading on the spring endpoints, these bonds can break or reform. The appropriate rates are obtained using the Bell model. These represent increasingly more accurate efforts to incorporate microscopic realism into the modeling. Finally, the simple model described here for an adherent cell in a shear flow ignores the deformability of the cell as well as any feedback between forces and shape. Any analytical description that accounts for deformability is challenging as fluid flow must be coupled to cell deformation.

### Shortcomings of Current Models

Several biologically relevant aspects need to be considered in developing better models. Velocities in arterial flows can sometimes be high enough for the fluid to enter a non-laminar regime, where the full Navier-Stokes equations are more appropriate than the simplified Stokes equations valid only in the *Re* → 0 limit. Biological fluids are also complex, structured and non-Newtonian, in general (Phillips et al., 2012). The simple boundary condition that is typically used ignores the role of the glycocalyx of the attached cell, a thin layer of extracellular membrane glycoproteins attached to the cell surface. It also ignores the possibility that the flow field can extend across the cell wall into the cytosol. The membrane also rotates around the cell body in what is termed as the tank-treading mode (Whitesides, 2006). Some calculations do treat the cell-fluid interface not as a solid-liquid interface as in the argument above but as an interface between two fluids of very different viscosities (Sugiyama and Sbragaglia, 2008).

The approximations that make the problem of cell detachment tractable analytically also ignore complexities of the shape of the spread cell, typically consisting of a flat portion from which the nucleus bulges out, resembling a fried egg. There are ways of generalizing the calculation indicated above to shapes that differ from the hemisphere. In this regard, Pozrikidis has provided numerical solutions for flows across protuberances above a surface that can be thought of as sections of oblate spheroids. These calculations indicate that the scaling of the total force with the contact area remains quadratic, as would be indicated purely by dimensional arguments. The precise geometry enters in the form of a geometrical pre-factor of order 1–10 (Pozrikidis, 1997; Hosoda et al., 2011).

The force-induced remodeling of adhesion complexes, torques experienced by the cell, and possible changes to cell shape due to shear stress are also usually ignored (Krendel et al., 2002; Kirchner et al., 2003; Ezratty et al., 2005; Wang and Dimitrakopoulos, 2006; Fletcher and Mullins, 2010; Wei et al., 2015). Finally, the cell itself can exhibit complex rheology or flow behavior at long times. Cells behave like liquid drops under surface tension at long times whereas at short times and for not too large deformations, the cell behaves like a solid elastic sphere. Both experiments as well as models describing cell detachment kinetics should also consider the exact mode of detachment. Cells can “peel-off” where bonds detach sequentially from one edge or can “lift-off” where the number of detached bonds increases uniformly over the cell area until the remaining bonds detach catastrophically. For these, considerations of the torque exerted by the flow on the adhered cell will also be important.

## Methods to Quantify Cell Adhesion

Several techniques have been used to measure cell adhesion strength. Of these, the most popular is the fluid shear flow method, where the fluid flows over a cell monolayer to apply a shear stress on cells. The applied shear stress can be adjusted to be in either of the following two regimes. At low fluid shear, the overall cell adhesion and cellular functions remain physiological and we can investigate the dynamic remodeling of cellular mechano-sensitive responses. In contrast, at higher shear stresses we can investigate the strength of the cell adhesions by monitoring cell detachment kinetics as a function of time (Maan et al., 2018). The detachment kinetics or remodeling dynamics due to shear stress is observed using multiple techniques. Microfluidic devices use fluid manipulation in channels with small dimensions (10–100 μm) and are generally fabricated using soft lithography which involves bonding of PDMS to glass that aid in the manufacturing of several near-identical devices (Xia and Whitesides, 1998; Whitesides, 2006). The device can be mounted on a microscope, facilitating real time experimentation (Lu et al., 2004). The advantage of this technique is that it provides well-characterized flow behavior and many parallel experiments can be done for high throughput experiments (Young et al., 2007). Adhesion studies using this method are done by varying either the protein coating on the substrate or the shear stress (Lu et al., 2004; Couzon et al., 2009). Young et al. designed a microfluidic device with eight identical parallel microchannels coupled to a single reservoir through a symmetric branched network. They measured the time profiles of cell-substrate adhesion strength by using different protein combinations and various levels of protein coating concentrations (Young et al., 2007).

Alternatively, shear stress can be applied to cells grown on a petri dish by placing a rotating cone-plate immersed in the medium just above the cells. This generates a rotational flow and the cone angle ensures a constant fluid shear stress at every point beneath the cone. Such devices use fluid shear stress over adherent cells cultured on substrates to study population averaged cell detachment kinetics (Khalili and Ahmad, 2015; Maan et al., 2018). This allows application of a wide range of forces to large cell populations that yield reliable measurements of cell adhesion strengths (Garcia et al., 1997). This method, while more difficult to construct, has a distinct advantage that it uses very little medium/reagents, is well-suited for long term experiments, and is also amenable to real-time high-resolution imaging (Maan et al., 2018). In one such study, Garcia et al. (1997) used spinning disc device consisting of a cylinder filled with fluid and sample disk to quantify cell adhesion. Using this device, they demonstrated the reproducibility and sensitivity in cell adhesion strengths by probing cellular adhesion to non-reactive and bioactive materials (Garcia et al., 1997, 1998). Maan et al. (2018) constructed a compact and microscope mountable device to apply shear stress to adherent cells. The study showed that the deadhesion profile of cells is dependent on the cell area (Figure 4A). Cells were subjected to shear stress using a 1° cone in the custom device. Stress was linearly increased, and time-lapse images of the cells were recorded to determine the number of adherent cells at each value of shear stress. The sigmoidal profile of the cell detachment curve under shear was used to calculate a critical value of shear stress for each cell type.

A simpler method to study differences in cell adhesion is to grow cells on plastic strips and subject them to centrifugation. In this case, force can be quantified if the density difference between the cell and the fluid medium is known. In this method, cells are seeded on the surface of a plastic strip and mounted perpendicular to the rotation axis such that the centrifugation force is tangential to the surface. In another geometry, a multi-well plate containing adhered cells is mounted such that the bottom surface of the multi-well plate is parallel to the axis of rotation so that the centrifugation force is normal to the surface. The number of adhered cells before and after centrifugation is quantified as an estimate of the adhesion strength (Hertl et al., 1984; Kevin et al., 2010). The remaining adhered cells after this assay can be determined by measuring the amount of radioactive emission from remaining radio labeled cells using a gamma counter (Giacomello et al., 1999; Koo et al., 2002), or by analyzing fluorescence with automatic machines (Channavajjala et al., 1997; Giacomello et al., 1999). The disadvantage of this technique is that imaging or real-time information cannot be obtained. Koo et al. (2002) used this method to show that ligand density and clustering are important in wild type NR6 fibroblasts which express αvβ3 and α5β1 integrins. Channavajjala et al. (1997) used this method to understand the significance of cell attachment to HIV- 1 Tat protein; showed a significant but weak attachment of Tat protein with HT1080 cells.

Reyas and Gracia described a modified centrifugation assay to quantify the adhesion strength of different cell lines on substrates. They showed that using different coating proteins showed increase in numbers of adherent cells after the assay with increasing initial cell adhesion time (Reyes and García, 2003). Kihara et al. (2018) quantified the adhesion strength of epithelial cells on a smooth titanium substrate. They showed that titanium treated with synthetic peptides (A 10 and PARA- AP) had stronger adhesion than non-treated titanium.

Another technique called laser catapulting uses an intensive shock wave induced by a laser beam on the cell which causes it to detach from the surface. The force exerted on a cell (>1 mN) by the pressure wave is greater than the force range achieved either by optical tweezer or by a magnetic tweezer. The advantage of this method is that the use of a short pulse duration in experiments means that cells do not have enough time to remodel and do not react to detachment force (Hu et al., 2006; Sada et al., 2011; Burk et al., 2015). Sada et al. (2011) used an NIR pulse laser (1,064 nm, 4 ns) to achieve selective detachment of cells cultured on SWNT (single-walled carbon nanotube) coated dish. This study showed the retention of the genetic information of the cell by PCR quantification. Burk et al. (2015) used this technique to determine the adhesion strength between human hematopoietic stem cells (HSC) and the bone marrow niche. A more complete overview of techniques can be found in a recent review by Khalili and Ahmad (2015).

## Conclusions

In this review, we have discussed the biological responses of cells subjected to shear stress, biophysical methods to quantify cell-substrate adhesions, and analytical approaches to model these responses. However, there are several open avenues for exploration that we identify. The theoretical formulation ignores several features of the biological system as discussed in a previous section on shortcomings of current models. More refined theoretical approaches are required to incorporate these features. Cells also remodel under shear, resulting in changes to cell shape, FAK signaling, and cytoskeleton. Only a few studies have quantified the corresponding cellular tractions at individual FA level for cells under shear. FRET based sensors to characterize strain sensing under shear are an attractive method when combined with the fluid shear device to probe the roles of different proteins that are involved in mechanosensing (Kumar et al., 2016).

Cell-substrate interactions play crucial roles in disease initiation and progression, tissue engineering, and in developmental biology. Several key molecules, such as vinculin, paxillin, and talin have been identified in mechanotransduction via the FA complex. The spatio-temporal events underlying changes in the activity of these proteins under mechanical stimuli are, however, not well-understood. Thus, measurements that integrate the application of controlled forces with measurements that probe the resulting cell response should be particularly valuable in the understanding of cellular and sub-cellular mechanobiology. Experiments using a fluid shear device can be used to visualize dynamic cellular changes in real-time and are useful in delineating the role of mechanosensing proteins. Quantifying the nature of cell-substrate adhesions under shear should also help in developing diagnostics for several diseases in which altered cell adhesion is a primary feature. Matrix remodeling by cancer cells during EMT to control adhesion strengths and modulate their migratory potential warrants careful examination using long term shear experiments. Biophysical models to quantify changes from slip to catch bond, the incorporation of stress fibers and calcium signaling under shear are also essential to advancing our understanding of how cell mechanosensing under fluid shear stress manifests itself at the level of cell adhesion.

## Author Contributions

PP and AM wrote the section related to experimental methods for cells under shear. GM wrote the section on biophysical models. NP and NG wrote the other sections in the manuscript with feedback from PK. NG oversaw the manuscript submission. All authors agree with the content in the manuscript.

### Conflict of Interest

The authors declare that the research was conducted in the absence of any commercial or financial relationships that could be construed as a potential conflict of interest.

## Abbreviations

ECM: extra cellular matrix
FA: focal adhesion
FAK: focal adhesion kinase
FSS: fluid shear stress
IF: intermediate filaments
LAP: latency associated protein
MKL1: megakaryoblastic leukemia factor-1
MMP: matrix metalloproteinase
MT: microtubules
RGD: Arg-Gly-Asp
ROCK: Rho and Rho kinase
EMT: epithelial–mesenchymal transition.
